# TMEM33, an oncogene regulated by miR-214-3p, promotes the progression of lung adenocarcinoma through the Wnt/β-catenin signaling pathway

**DOI:** 10.32604/or.2024.052089

**Published:** 2025-03-19

**Authors:** GUANGXIAN YOU, QIAO YANG, XIN LI, LILI CHEN

**Affiliations:** 1 Graduate School, Zhejiang Chinese Medical University, Hangzhou, 310053, China; 2 Department of Hematology and Oncology, Taizhou First People’s Hospital, Taizhou, 318020, China

**Keywords:** Lung adenocarcinoma (LUAD), Transmembrane protein 33 (TMEM33), miR-214-3p, Wingless (Wnt), Malignant progression

## Abstract

**Background:**

Lung cancer remains a major factor causing cancer-associated mortality globally. While there have been advancements in treatment options, advanced lung cancer patients still have poor outcomes. This study aims to investigate the potential role of Transmembrane protein 33 (TMEM33) in the development of lung adenocarcinoma.

**Methods:**

We leveraged The Cancer Genome Atlas (TCGA) database to analyze the connection between TMEM33 expression to the prognosis of lung adenocarcinoma (LUAD). Cell proliferation, invasiveness, and sphere formation were analyzed by various experiments. The association of miR-214-3p with TMEM33 was explored using luciferase reporter assay, immunoblotting, and real-time quantitative PCR (RT-qPCR). Additionally, TMEM33’s biological role was confirmed in the mouse xenograft model through lung cancer transplantation and metastasis studies.

**Results:**

TMEM33 showed high expression within both LUAD tissues and cells, with its expression correlating with poor patient survival outcomes. Silencing TMEM33 resulted in significant reductions in cell proliferation, invasiveness, and stem-like properties. Further investigation suggested that miR-214-3p negatively regulated TMEM33. In both cellular and animal models, we further demonstrated that TMEM33 knockdown could effectively suppress the aggressiveness of lung cancer cells, impeding tumor growth and inhibiting metastasis in the mouse model. Moreover, reducing TMEM33 expression reduced key signaling molecules within the Wnt/β-catenin pathway, providing insights into TMEM33’s mechanistic role in LUAD.

**Conclusion:**

TMEM33 functions as an oncogene, which is under the negative regulation of miR-214-3p, to promote the LUAD malignant characteristics by engaging the Wnt/β-catenin cascade.

## Introduction

Lung cancer is a major factor inducing cancer-associated mortality globally, and more than one million new cases and around 600,000 fatalities—an escalating figure annually are reported [1,2]. Predictions indicate a potential surge to 25 million fresh instances of lung cancer by 2035 [3]. Significantly, roughly one-quarter of patients receive a late diagnosis at Stage III or IV, rendering conventional treatments ineffective and resulting in high mortality rates [4]. Additionally, recurrence occurs in nearly half of early-stage patients [5]. Despite the advancement of therapeutic options, such as targeted and immunotherapies in combination with conventional surgical and radiotherapeutic methods, survival rates for advanced lung cancer are disappointingly low [3]. This highlights the urgent requirement to uncover novel molecular mechanisms governing lung cancer, leading to the identification of clinically significant biomarkers and treatment targets.

TMEM33 (transmembrane protein 33) is known as an oncogene with elevated levels present in cervical and renal cancers [6–9]. Although its involvement in lung cancer has not yet been documented, previous research has suggested that various TMEM family proteins, such as TMEM168, TMEM9A, TMEM48, and TMEM14A, are important for regulating malignant tumor characteristics through impacting Wnt/β-catenin pathway [10–13]. Nevertheless, how TMEM33 overexpression impinges on the aggressiveness of lung cancer cells and tumor progression has not yet been explored.

MiRNAs represent small RNAs essential for regulating genes. They bind to 3′-UTRs in target mRNAs to reduce mRNA levels, impacting various biological functions. Non-coding RNAs, especially miRNAs, exert crucial effects on cancer studies. They can either promote or inhibit cancer by controlling important genes in cellular pathways. miRNAs are vital for tumor occurrence and spread [14]. They are demonstrated with important effects on cancer behavior and advancement. In lung cancer research, miRNAs have been associated with diagnosis, progression, and treatment response. For example, miR-584-5p is linked to prognosis and regulates RAB23 to affect lung inflammation in LUAD [15]. The inhibition of miR-33b-5p targets YWHAH to promote chemoresistance, impacting EMT in LUAD [16]. This suggests that focusing on miRNAs in research could lead to innovative approaches for early detection and treatment of LUAD.

Among the putative regulatory miRNAs, only miR-214-3p could modulate TMEM33 expression as supported by existing literature [17–19]. miR-214-3p shows down-regulation within lung cancer, where it plays the role of the tumor suppressor [17–19]. miR-214-3p down-regulation within ovarian cancer impedes cancer cell metastasis by targeting Nuclear Paraspeckle Assembly Transcript 1 (NEAT1) [20]. Furthermore, abnormal expression of miR-214-3p within cervical cancer is associated with tumor cell migration through its interplay with Thrombospondin 2 (THBS2) [21].

The current research was conducted to explore the biological significance of TMEM33 and the mechanisms associated with LUAD progression. The present work comprehensively assessed TMEM33 expression patterns, their clinical implications, and their effects on cancer cell proliferation, invasion, and stemness using samples from patients, cell cultures, and animal models.

## Materials and Methods

### Tissue sample extraction

Tissue samples were obtained from 86 cases of LUAD and corresponding non-tumorous tissue at Taizhou First People’s Hospital from 2019 Nov–2023 Oct and preserved within liquid nitrogen at once. The collection of samples used in this study was approved by the Ethics Committee of Taizhou First People’s Hospital (Approval number: 2023DL057). Informed consent was obtained from all patients from whom tissue samples were taken. The inclusion criteria were: (1) diagnosis of LUAD according to established criteria; (2) surgical treatment with a confirmed pathological diagnosis (independent confirmation by two pathologists) from the Department of Pathology. Exclusion criteria were: (1) metastatic lung cancer, large or small cell lung cancer, or squamous cell carcinoma; (2) adenocarcinoma with mixed cancer types.

### Cell culture and transfection

We obtained lung cancer cells (A549, H1299, PC9, and H1975) and BEAS-2B normal human bronchial epithelial cells from Typical Cultures Preservation Center of Chinese Academy of Sciences (Shanghai, China). All cells were authenticated by STR profiling and tested to be mycoplasma-free. These above-mentioned cells were cultured under 37°C with 5% CO_2_, with Dulbecco’s Modified Eagle’s Medium (Gibco, GrandIsland, MA, USA) that contained 10% fetal bovine serum (Gibco, GrandIsland, MA, USA), 1% penicillin, and streptomycin (100 U/mL P/S; Gibco, MA, USA). Furthermore, sh-TMEM33#1/2/3, miR-214-3p mimics, and miR-214-3p inhibitor, together with corresponding controls were provided by GenePharma (Shanghai, China). To enhance TMEM33 expression, the pcDNA 3.1-TMEM33 vector was prepared. Transfection was carried out with Lipofectamine 3000 reagent (Invitrogen, Gibco, GrandIsland, MA, USA) per specific protocols. Logarithmic-phase cells were inoculated into 6-well plates at 2.5 × 10^5^ cells/per well and transfected with 3 μg of the target gene plasmid or other materials once they reached 80% confluence. At 48-h after incubation in 5% CO_2_, transfected cells were utilized for subsequent experiments.

### RNA isolation and RT-qPCR

TRIzol reagent (Invitrogen; Gibco, GrandIsland, MA, USA) was utilized for extracting total RNA. The RNA concentration was determined by measuring the absorbance at 260 nm (A260) with an enzyme-labeled instrument (model ELX800, Shanghai, China) and prepared into cDNA with the reverse transcription kit (Thermo Fisher, Waltham, MA, USA) through reverse transcription. For reverse transcription from miR-214-3p, the stem-loop based method was used, with the following stem-loop RT primer: 5′-GTCGTATCCAGTGCGTGTCGTGGAGTCGGCAATTGCACTGGATACGACACTGCC-3′.

Quantitative PCR (qPCR) was carried out using a SYBR Premix Ex Taq kit (Takara, Osaka, Japan) with the Applied Biosystems 7500 detection system. The qPCR procedure followed the manufacturer’s guidelines: 5 min initial denaturation under 95°C, then 5 s denaturation under 95°C and 30 s annealing under 60°C, with GAPDH and U6 being the endogenous controls for gene and miRNA expression normalization, separately. The 2^−ΔΔCt^ approach was employed for determining gene expression. Shanghai Sangong Bioengineering Co., Ltd. (Shanghai, China) was responsible for producing all qPCR primers: TMEM33 forward primer 5′-ACGCAAGGGGGCTCAAATAGT-3′; TMEM33 reverse primer 5′-TGTCGCAGGCATCAGGAATA-3′; miR-214-3p forward primer 5′-GCGACAGCAGGCACAGACA-3′; miR-214-3p reverse primer 5′-AGTGCAGGGTCCGAGGTATT-3′; GAPDH forward primer 5′-GCACCGTCAAGGCTGAGAAC-3′; GAPDH reverse primer 5′-TGGTGAAGACGCCAGTGGA-3′; U6 forward primer 5′-CTCGCTTCGGCAGCACA-3′; U6 reverse primer 5′-AACGCTTCACGAATTTGCGT-3′.

### Western blot assay

Protein content extraction involved the use of pre-chilled RIPA lysis buffer (BiyunTian Biologicals, Shanghai, China), with protein content analysis conducted with a Bicinchoninic Acid Protein Assay Kit (BiyunTian Biologicals, Shanghai, China). Following extraction, proteins underwent separation via electrophoresis on a 10% SDS-PAGE gel, prior to transfer on PVDF membranes. After blocking using 5% defatted milk for 1 h, membranes were subjected to primary antibodies targeting TMEM33 (Ab184164, 1:1000 dilution), β-catenin (Ab32572, 1:1000 dilution), c-Myc (Ab32072, 1:1000 dilution), Cyclin D1 (Ab16663, 1:1000 dilution), and GAPDH (Ab8245, 1:5000 dilution, Abcam, Massachusetts, USA). Post-primary antibody exposure, HRP-labeled secondary antibodies were applied for a 2 h period under ambient temperature before visualization of protein bands using an ECL detection system on a Gel-Doc 200 imaging system (Bio-Rad, Hercules, CA, USA), with GAPDH being the endogenous control for protein expression normalization.

### Dual-luciferase reporter assay

The *TMEM33* 3′-UTR wild-type (WT) sequence, which contained the candidate miR-214-3p binding site, was subjected to PCR amplification (Forward primer 5′-AGGTTTGGGAGGTTTACTGGT-3′, Reverse primer 5′-CCTACACGCACAAGAGGAGA-3′) and insertion in the pmirGLO luciferase reporter vector (Promega, Madison, WI, USA). Mutant (Mut) sequence *TMEM33* 3′-UTR was generated using site-directed mutagenesis of the WT *TMEM33* 3′-UTR in the pmirGLO vector. TMEM33-WT or TMEM33-Mut constructs were co-transfected with either miRNA negative control (miR-NC) or miR-214-3p mimic in cells using Lipofectamine 3000 reagent. Luciferase activities were evaluated at 48 h post-transfection with a Dual-Luciferase Reporter Assay System (Promega, Madison, WI, USA). Firefly luciferase readings were normalized to the Renilla luciferase readings to determine relative luciferase activity with the formula: normalized value = Firefly luciferase reading/Renilla luciferase reading.

### Cignal™ finder cancer 10-pathway reporter array

The activation status of ten unique cancer-related signaling pathways in LUAD cells was analyzed using luciferase reporter assays with Cignal™ Finder 10-Pathway Reporter Array (Qiagen, Düsseldorf, Germany) in line with provided instructions. Luciferase activities were measured utilizing the Dual-Luciferase Reporter Assay System (Promega, Madison, WI, USA).

### CCK-8 assay

Cell proliferation was analyzed by Cell Counting Kit-8 (CCK-8; Dojindo, Kyushu Island, Japan) following specific protocols. Initially, 3000 cells per transfected group were plated in 96-well plates, with six replicates per group. Subsequently, after the incubation for 0/24/48/72 h, CCK-8 solution (10 μL) was added into every well for a further 3 h incubation under 37°C. Optical densities at 450 nm were then quantified with the enzyme labeling instrument (model ELX800, Shanghai, China).

### EdU assay

EdU cell Proliferation Kit (Abcam, Boston, MA, USA) was utilized for evaluating cell proliferation. Briefly, 5 × 10^5^ cells were incubated in a fresh medium containing 40 mM EdU for 3 h at 37°C. Later, 200 μL of fixative was added to each well, with subsequent shielding of the cells from light for a 15-min duration under ambient temperature. This step was succeeded by permeabilization for 10 min and staining with Hoechst 33342 for 30 min at room temperature. Stained cells were visualized with the fluorescence microscope from Olympus in Tokyo, Japan.

### Sphere formation assay

LUAD cell groups, including both control and experimental conditions, were evenly distributed into ultra-low attachment microplates with round bottoms (Corning Incorporated, Corning, NY, USA) at 1 × 10^5^ cells/mL, followed by 1-week incubation under 37°C. After incubation, the diameter of the spheres was determined and quantified by utilizing a light microscope from Olympus in Tokyo, Japan. This procedure was replicated three times, with each repetition consisting of three biological duplicates.

### Transwell invasion assay

Following a 24-h period of starvation, cells were harvested for the Transwell test. The inserts used in the Transwell test were coated in Matrigel (Yeason, Shanghai, China) to mimic the extracellular matrix. After suspension within a serum-free culture medium, cells were added in the top well of the Transwell compartments with 5 × 10^4^ cells in each well, while the bottom part was introduced DMEM containing 10% serum to attract the cells. Following 24 h culturing, the cells remaining on the top well were eliminated, whereas the invading cells on the bottom surface were fixed with 4% paraformaldehyde prior to 0.1% crystal violet solution staining and observation with the light microscope.

### Animal studies

Female nude mice (four-week-old, n = 20) were obtained in Shanghai Model Biological Center in Shanghai, China. Each group consisted of five mice that were injected subcutaneously with cell suspensions of A549 cells (5 × 10^6^ cells per animal) subjected to sh-NC or sh-TMEM33 transfection. Tumor volume was determined at 5-day intervals by V = 0.5 × L × W^2^, in which L and W represent long and short tumor axes, separately. Following this, euthanasia was conducted in animals through cervical dislocation, then tumor tissues were collected for weight measurement. Furthermore, A549 cells (5 × 10^6^ cells per animal) with stable transfection of sh-NC or sh-TMEM33 were injected in nude mouse tail veine, with each group consisting of five mice. At eight weeks later, euthanasia was conducted in animals, later, the lung nodules were collected for both counting and histological examination through hematoxylin and eosin (HE) staining. All procedures involving animals were conducted in accordance with the guidelines and approval of the Taizhou First People’s Hospital Ethics Committee (2023DL057). The study is reported in accordance with ARRIVE guidelines (https://arriveguidelines.org, accessed on 27 February 2023).

### HE staining

The lung tissue samples underwent systematic deparaffinization and rehydration, followed by being stained with hematoxylin for a duration of 3 min. Next, the tissue sections were placed in 0.5% hydrochloric acid alcohol solution for 30 s, followed by exposure to a series of alcohol gradations, and then stained with 0.5% eosin for 1 min. Following additional dehydration steps using alcohol gradations and clearing with xylene, samples were mounted with neutral gum before the examination using an Olympus BX53 light microscope (Olympus Corporation, Tokyo, Japan).

### Immunohistochemical staining

Immunohistochemical staining was performed on the sections of xenograft tumors after a series of preparatory steps including baking, deparaffinization, and antigen retrieval. Initially, the sections were blocked with a solution of 5% goat serum and put into the humidified chamber under 37°C for 30 min. Primary antibodies against β-catenin, TMEM33, Ki-67, or SOX2 from Abcam (Boston, MA, USA) were applied at 1:100, and later incubated under 4°C overnight. The next day, the sections were subjected to a 45-min incubation at 37°C. Subsequently, an HRP-conjugated sheep anti-rabbit secondary antibody from Abcam (Boston, MA, USA; 1:1000) was added to incubate sections under 37°C for a 1 h period. After washing, sections were exposed to a DAB solution (a mixture of DAB1 and DAB2 in a 1:20 ratio) for 5 min while shielded from light, and then counterstained with hematoxylin for 5 min. Following washing and dehydration in ethanol, the sections were mounted for observation under a microscope.

### Analysis of public databases

The levels of TMEM33 in LUAD tumor samples and neighboring non-neoplastic lung tissues were studied utilizing data based on the TCGA-LUAD database (https://portal.gdc.cancer.gov/projects/TCGA-LUAD, accessed on 13 September 2018 and confirmed on 06 March 2023). The GEPIA platform (http://gepia.cancer-pku.cn/, accessed on 13 September 2018 and confirmed on 06 March 2023) was adopted for analysis. Furthermore, TMEM33 expression in LUAD was also assessed via the KM plotter online resource (http://kmplot.com/analysis/, Affy ID: TMEM33, accessed on 13 September 2018 and confirmed on 06 March 2023). This investigation enabled the comparison of survival rates among groups of patients with varying levels of TMEM33 expression.

### Statistical analysis

Mean ± SD values were reported for all experimental findings, with a minimum of three independent trials conducted. Data were analyzed with GraphPad Prism 6.0 software (GraphPad Software, Boston, NY, USA). Between-group differences were compared with Student’s *t*-test, while among-group differences were compared by ANOVA. Relation of expression levels was evaluated by Spearman correlation coefficient analysis. Kaplan-Meier curves were created for survival analysis and the log-rank test was adopted to conduct significance determination (Affy ID: TMEM33). We utilized the Chi-square test to investigate relations of categorical data. *p* < 0.05 stood for a significant difference.

## Results

### TMEM33 overexpression within LUAD tissues and cells

**Figure 1:**
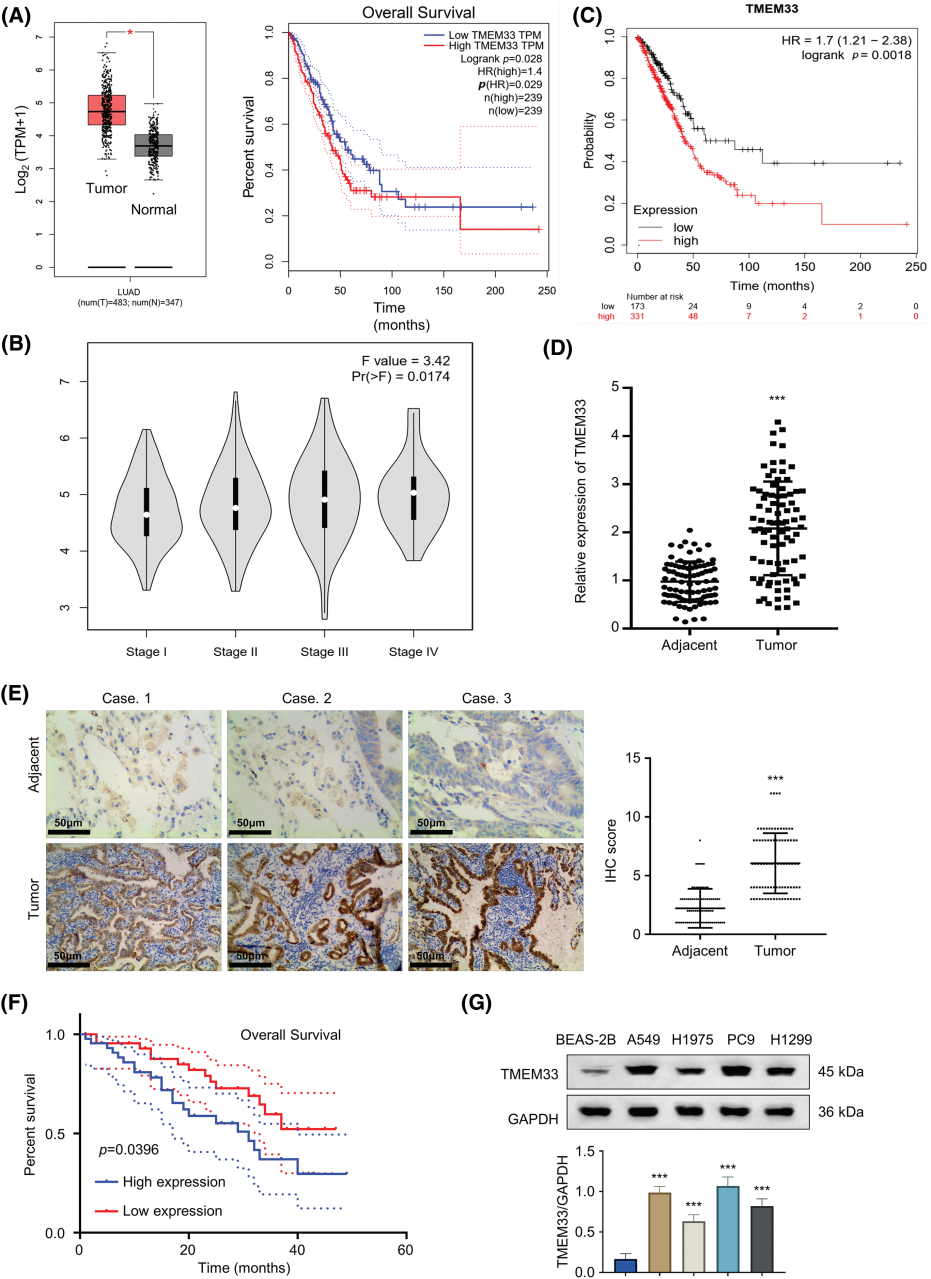
TMEM33 is highly expressed in LUAD tumor tissues and cell lines. (A) Analysis of TMEM33 expression in LUAD tissues and normal lung tissues from the TCGA-LUAD database (left), and analysis of the difference in overall survival of these cases grouped by high and low TMEM33 expression (right). (B) Differences in TMEM33 expression in patients with different stages. (C) Kaplan-Meier analysis of overall survival in patients with high TMEM33 expression *vs*. low TMEM33 expression in LUAD (Affy ID: TMEM33). (D) The expression of TMEM33 in 86 pairs of LUAD tissues and normal lung tissues was verified by qRT-PCR. (E) The expression of TMEM33 in LUAD tissues and normal lung tissues was verified by immunohistochemical staining (scale bar = 50 μm). (F) Kaplan-Meier analysis of overall survival in patients with TMEM33 high-expression (n = 43) *vs*. low-expression LUAD (n = 43). (G) Expression of TMEM33 in 4 LUAD cell lines and BEAS-2B cell line was analyzed by Western blot. *p < 0.05, and ***p < 0.001.

Our exploration of the TCGA-LUAD database via the GEPIA online platform showed that TMEM33 showed up-regulation within LUAD tissues, which was associated with decreased patient overall survival (OS) (Fig. 1A). Furthermore, data from the same database revealed that TMEM33 expression increases with the progression of LUAD grade (Fig. 1B). These findings were further supported by the analysis using the KM plotter (Affy ID: TMEM33), which indicated that patients with heightened TMEM33 expression suffered from a poorer OS (Fig. 1C). To verify these results, we analyzed 86 LUAD tumors together with 86 matched non-carcinoma samples, confirming overexpression of TMEM33 in the cancerous tissues through RT-qPCR (Fig. 1D). The overexpression of TMEM33 was also confirmed in 20 randomly selected pairs of LUAD and matched non-carcinoma tissues by IHC (Fig. 1E). Those 86 LUAD patients were divided into two groups according to median TMEM33 expression, including the low- and high-expression groups (n = 43 each). Conforming to database analysis, patients showing TMEM33 high-expression showed dismal prognoses (Fig. 1F). Additionally, a chi-square test exploring the relation of TMEM33 with various clinicopathological features showed a significant correlation with lymph node metastasis, TNM stage, and tumor differentiation, rather than with age, sex, and tumor size (Table 1). Apart from tissue samples, TMEM33 expression was evaluated in four LUAD cells (A549, H1299, PC9, and H1975) and BEAS-2B cells. These findings revealed consistently higher TMEM33 expression in all tested LUAD cell lines (Fig. 1G). Overall, these results suggest TMEM33 up-regulation within LUAD tissues and cells.

**Table 1:** Associations of LUAD clinicopathological characteristics with TMEM33 expression

Characteristics	Total number	TMEM33 expression		*p*-value
		Low (n = 43)	High (n = 43)	
Age				0.5102
<55	35	19	16	
≥55	51	24	27	
Gender				0.5135
Male	49	23	26	
Female	37	20	17	
Smoking				0.2788
Yes	39	22	17	
No	47	21	26	
Tumor size				0.0512
<5 cm	47	28	19	
≥5 cm	39	15	24	
TNM stage				0.0172
Stage I/II	47	29	18	
Stage III/IV	39	14	25	
Lymph node metastasis				0.046
Negative	53	31	22	
Positive	33	12	21	
Tumor location				0.5135
Left lung	37	20	17	
Right lung	49	23	26	
Survival years after surgery				0.052
<3 year	45	18	27	
≥3 year	41	25	16	

### Inhibition of LUAD cell proliferation, invasion, and stemness by TMEM33 knockdown

**Figure 2:**
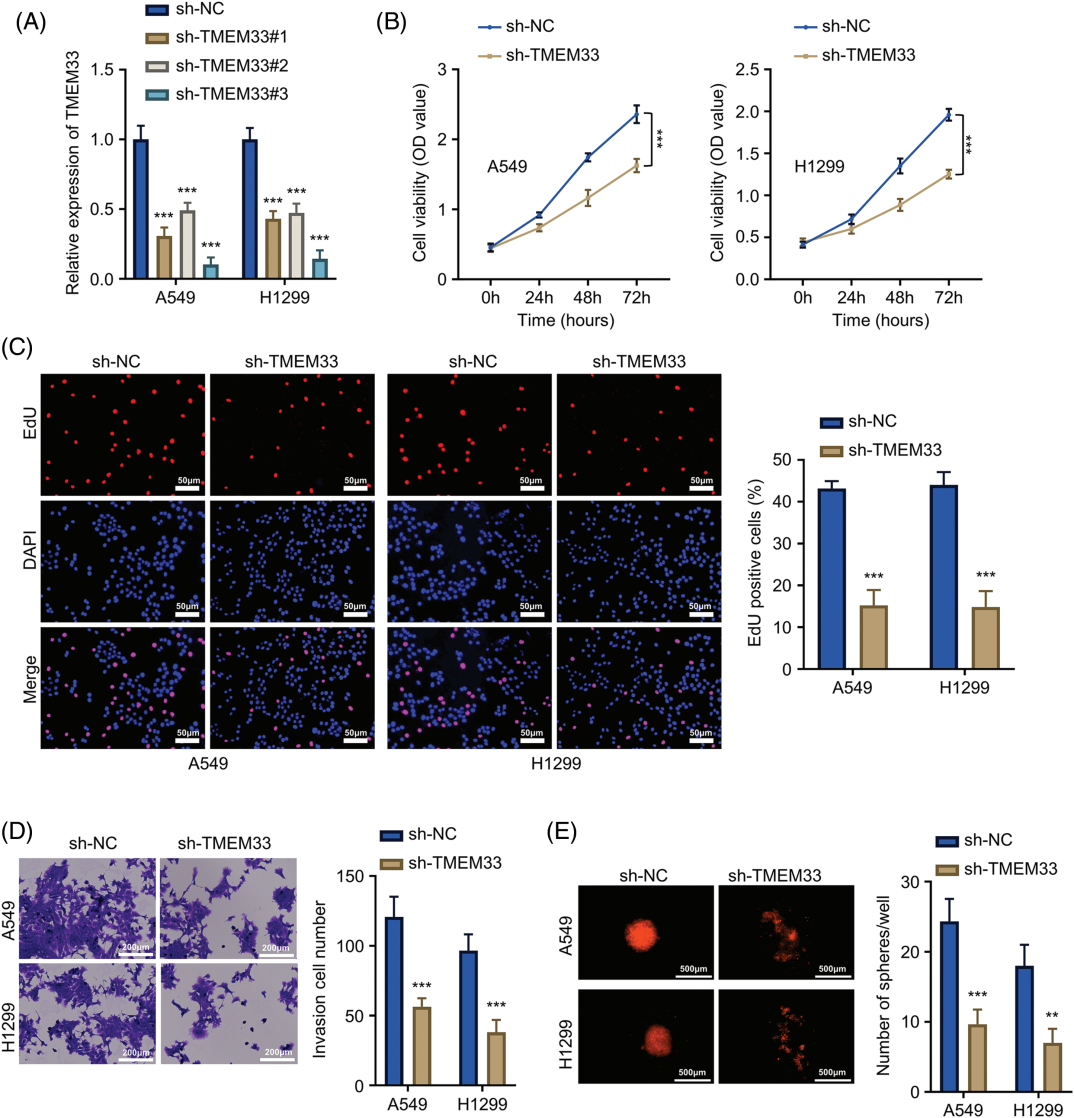
Knockdown of TMEM33 inhibits proliferation, infiltration, and stemness characteristics of LUAD cells. (A) The knockdown efficiency of shTMEM33#1/2/3 in A549 and H1299 cells was verified by qRT-PCR. (B–E) The effects of the knockdown of TMEM33 on cell proliferation by CCK-8 assay (B) and EdU incorporation assay, scale bar = 50 μm (C), and the effects of TMEM33 knockdown on the invasive ability, scale bar = 200 μm (D) and sphere formation, scale bar = 500 μm (E) were verified in A549 and H1299 cells. **p < 0.01; ***p < 0.001.

For exploring TMEM33’s effect on LUAD cell aggressiveness, this study constructed stable TMEM33 knockdown cell lines using A549 and H1299 cells with relatively high expression of TMEM3. Among different shRNAs, sh-TMEM33 #2 exhibited the most efficient silencing of TMEM33 and was used for additional investigation (referred to as sh-TMEM33) (Fig. 2A). Initially, we assessed the impact of TMEM33 on the proliferative capabilities of these cells. TMEM33 silencing resulted in a notable decrease in cell proliferation, as revealed by CCK-8 and EdU proliferation assays (Fig. 2B,C). Additionally, the effects of TMEM33 knockdown on invasive and stem cell-like characteristics of the cells were analyzed. According to the results, TMEM33 silencing markedly undermined abilities to invade matrigel-coated membrane (Fig. 2D) and generate spheroids (Fig. 2E). Together, these findings suggest that lowering TMEM33 expression effectively impeded LUAD cell growth, invasion, and stem-like features.

### miR-214-3p targets and downregulates TMEM33 within LUAD cells

**Figure 3:**
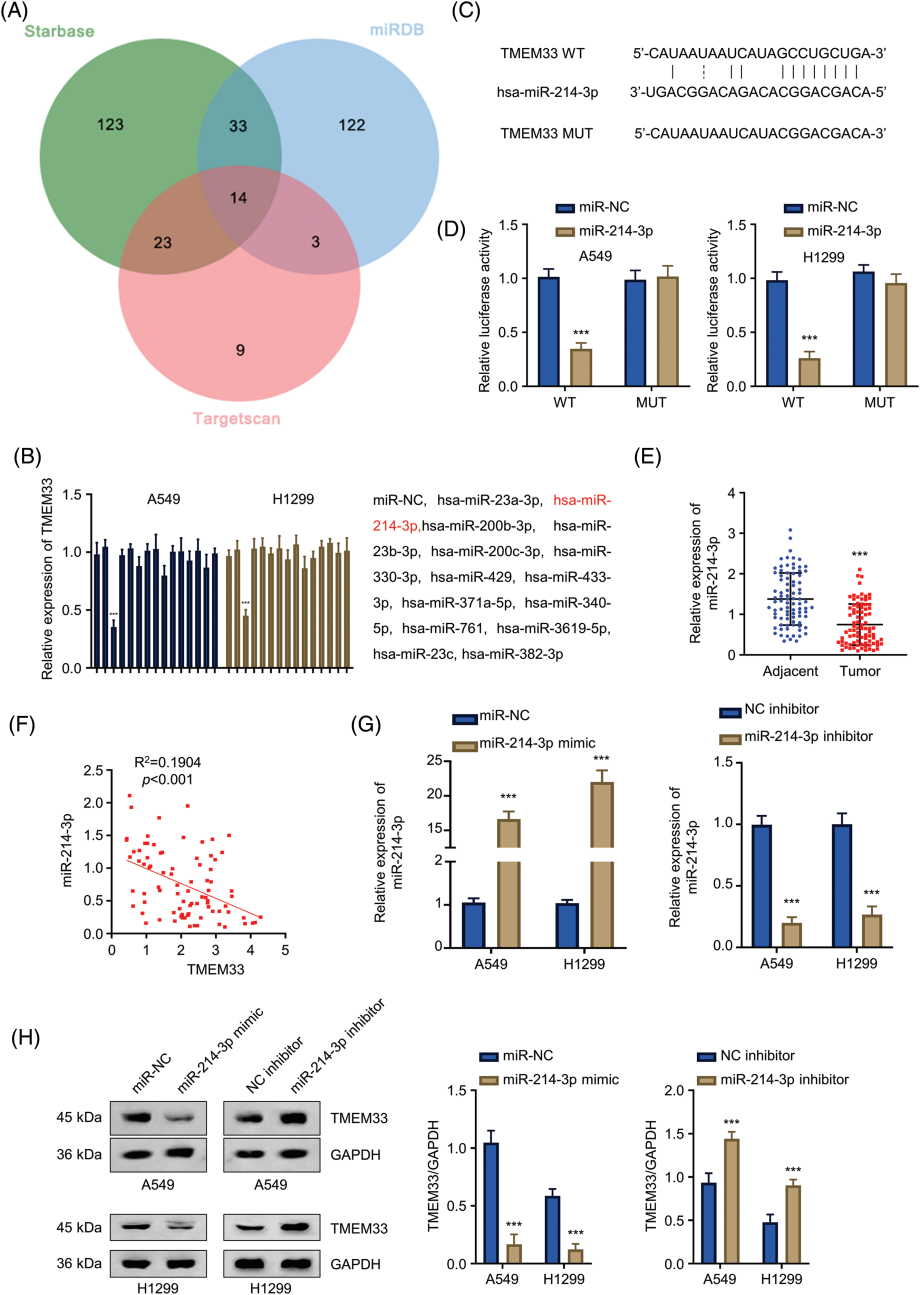
miR-214-3p can target TMEM33 and negatively regulate TMEM33 expression in LUAD cells. (A) miRNAs that may interfere with TMEM33 expression were predicted by “miRDB”, “StarBase” and “Targetscan” databases. (B) qRT-PCR was performed to detect the changes of *TMEM33* expression in A549 and H1299 cells upon overexpression of 14 miRNAs, respectively. The name of all miRNAs (from left to right) was specified by the bar graph. (C) Database-predicted binding of miR-214-3p to the WT *TMEM33* 3′-UTR and MUT *TMEM33* 3′-UTR sequences. (D) Interaction between miR-214-3p and the 3′-UTR of *TMEM33* was verified by luciferase reporter assay in A549 and H1299 cells. (E) The expression of miR-214-3p in LUAD tissues and normal lung tissues was verified by qRT-PCR. (F) Correlation analysis of miR-214-3p and TMEM33 expression in LUAD tissues. (G) The expression of miR-214-3p was detected in A549 and H1299 cells following either overexpression or inhibition of miR-214-3p. (H) The effects of miR-214-3p mimics or miR-214-3p inhibitors on TMEM33 expression in A549 and H1299 cells were detected by immunoblotting. ***p < 0.001.

MicroRNAs (miRNAs) are important for cancer biology by controlling target gene expression. Various miRNAs, including miR-23b-3p, hsa-miR-200c-3p, hsa-miR-330-3p, hsa-miR-429, hsa-miR-433-3p, hsa-miR-371a-5p, hsa-miR-340-5p, hsa-miR-761, hsa-miR-3619-5p, hsa-miR-23c, hsa-miR-382-3p (shown in Fig. 3A), were predicted as the putative regulators of TMEM33. RT-qPCR experiments were conducted after overexpressing 14 candidate miRNAs using miRNA mimics, and miR-214-3p significantly suppressed TMEM33 mRNA levels (Fig. 3B). Based on Starbase database prediction, miR-214-3p interacted with 3′-UTR in TMEM33 mRNA (Fig. 3C). Experimental validation confirmed this prediction, as overexpression of miR-214-3p reduced intracellular luciferase activity after transfection using WT reporter containing predicted binding sites, an effect reversed upon putative binding site mutation within MUT reporter (Fig. 3D). Additionally, miR-214-3p down-regulation could be observed within LUAD tissues in comparison with non-carcinoma counterparts (Fig. 3E). Analysis using the Spearman correlation coefficient (R = 0.4363, *p* < 0.001) revealed that miR-214-3p was significantly negatively correlated with TMEM33 expression within LUAD samples (Fig. 3F). Besides, upon the transfection using miR-214-3p mimic to elevate miR-214-3p levels, or the transfection with miR-214-3p inhibitor to suppress miR-214-3p (Fig. 3G), TMEM33 protein levels showed a notable decrease or increase within A549 and H1299 cells (Fig. 3H). Collectively, miR-214-3p targets TMEM33 and adversely modulates the expression within LUAD.

### miR-214-3p suppresses TMEM33-mediated enhancement of proliferation, invasiveness, and stemness within LUAD cells

**Figure 4:**
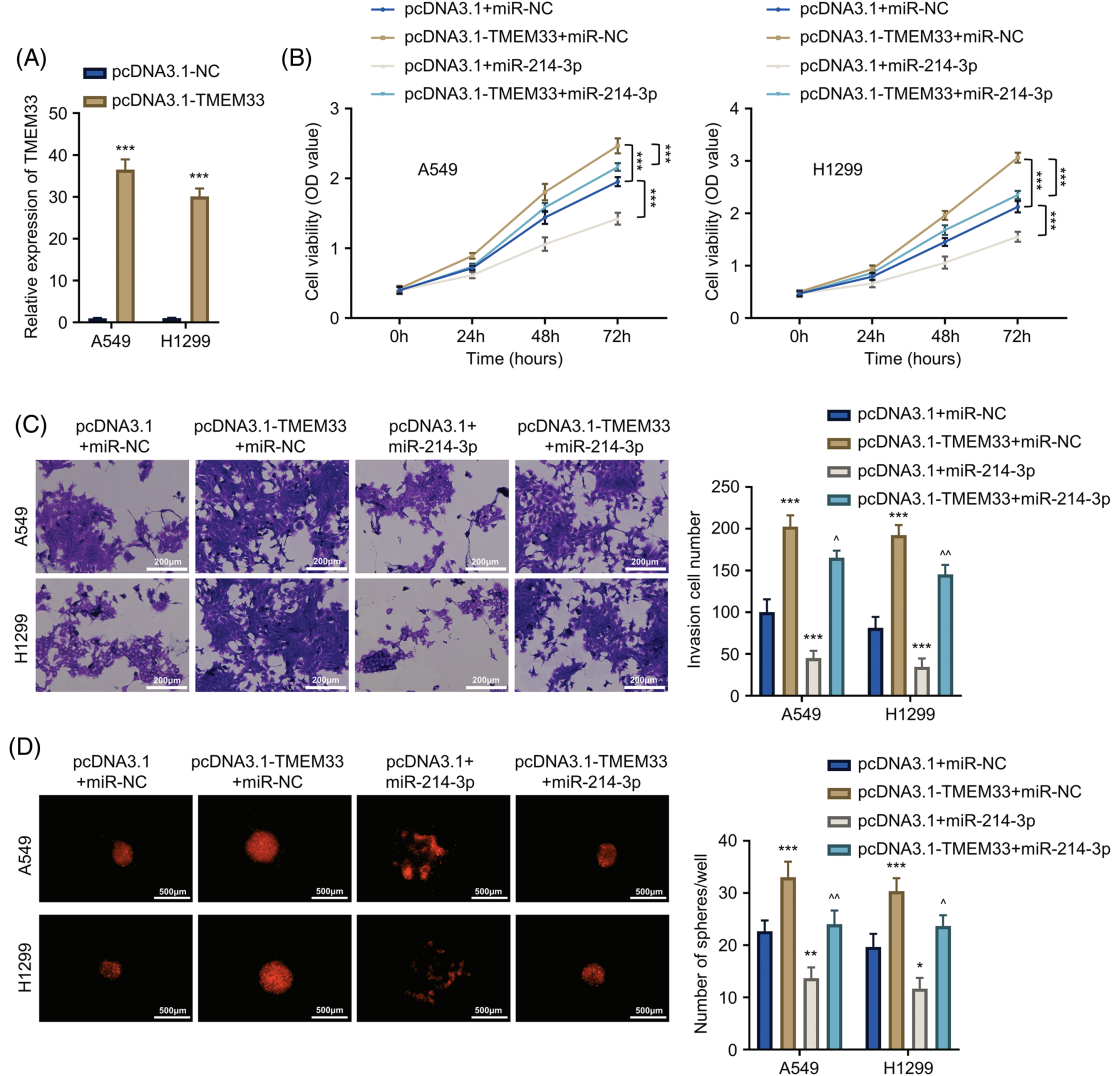
miR-214-3p partially reverses the promotional effect of TMEM33 overexpression on proliferation, metastasis, and stemness characteristics of LUAD cells. (A) The expression of TMEM33 in A549 and H1299 cells transfected with the control vector or pcDNA3.1-TMEM33 vector. (B–D) Confirmation of the impact of TMEM33 overexpression on cell growth, invasiveness (scale bar = 200 μm), and sphere-forming abilities (scale bar = 500 μm) in A549 and H1299 cells, with and without the presence of miR-214-3p. *p < 0.05, **p < 0.01, and ***p < 0.001 compared with pcDNA3.1+miR-NC group. ^^^*p* < 0.05 and ^^^^*p* < 0.01 compared with pcDNA3.1-TMEM33+miR-NC group.

To explore the regulatory interplay between miR-214-3p and TMEM33, we investigated how miR-214-3p affected LUAD cells after TMEM33 overexpression. Through successful TMEM33 overexpression within A549 and H1299 cells using the pcDNA3.1-TMEM33 plasmid (Fig. 4A), we co-transfected miR-214-3p mimic in cells. Cell proliferation of the pcDNA3.1-TMEM33+miR-NC group was remarkably enhanced relative to the pcDNA3.1+miR-NC group. Conversely, proliferation in the pcDNA3.1+miR-214-3p group markedly decreased in comparison with the pcDNA3.1+miR-NC group. Interestingly, the proliferation of the pcDNA3.1-TMEM33+miR-214-3p group showed a partial reduction compared to the pcDNA3.1-TMEM33+miR-NC group (Fig. 4B). Furthermore, the augmented invasive abilities (Fig. 4C) and spheroid formation (Fig. 4D) induced by TMEM33 overexpression were impaired by miR-214-3p mimic co-transfection. Based on the above findings, miR-214-3p can influence TMEM33 expression and modulate LUAD cell malignant progression *in vitro*.

### TMEM33 modulates proliferation, invasion, and stemness in LUAD cells via Wnt/β-catenin pathway regulation

**Figure 5:**
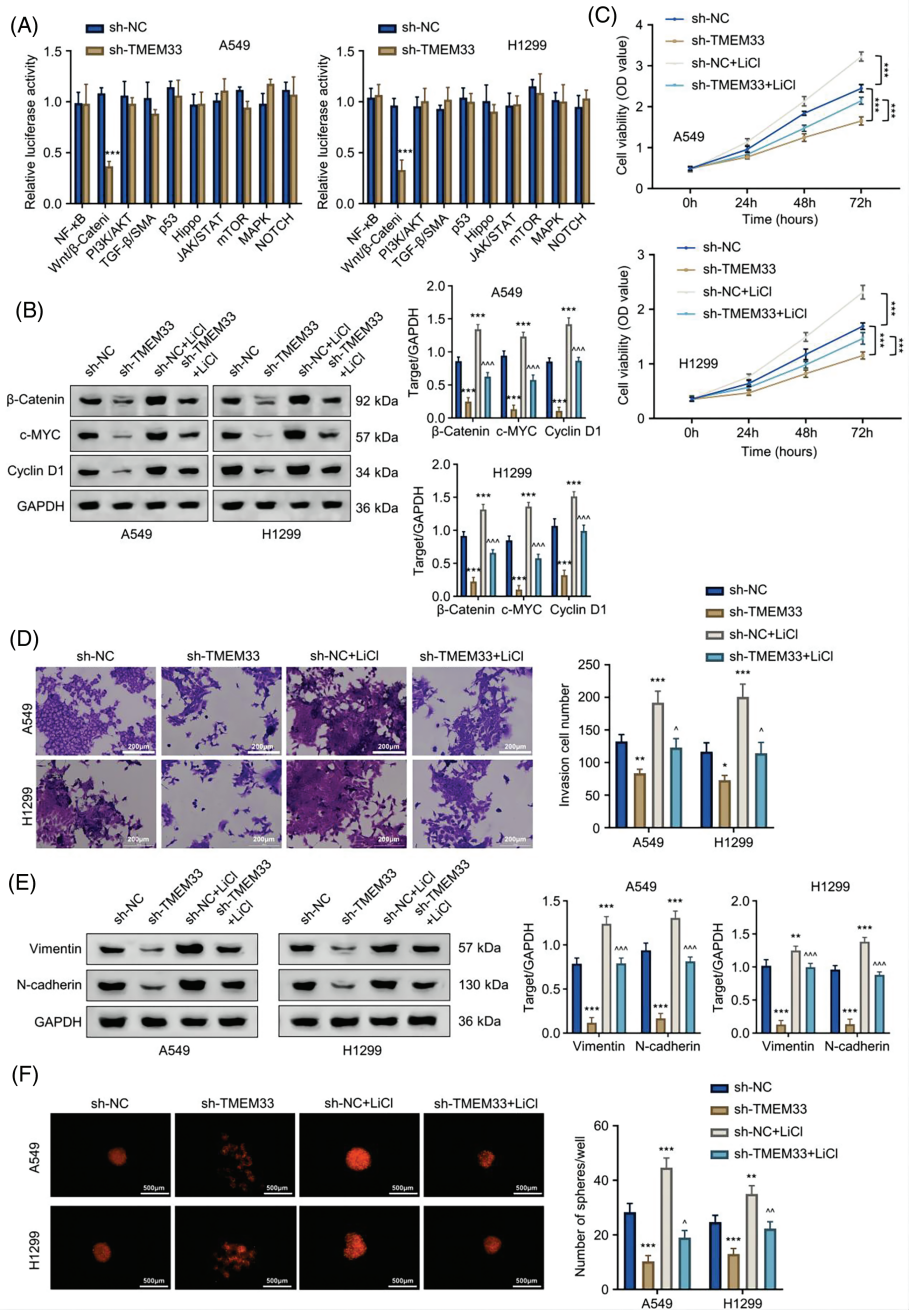
TMEM33 modulates the proliferation, metastasis, and sphere formation of LUAD cells by targeting the Wnt/β-catenin pathway. (A) The effect of TMEM33 knockdown on the activity of 10 signaling pathways was detected in A549 and H1299 cells. (B) Immunoblotting was performed to detect the effect of TMEM33 knockdown on the expression of Wnt pathway-related molecules (β-catenin, c-MYC, and Cyclin D1) in the presence or absence of LiCl-induced activation. (C) Detection of cell growth, (D) invasiveness (scale bar = 200 μm) (E) mesenchymal markers (Vimentin and N-cadherin) and (F) sphere-forming potential (scale bar = 500 μm), with or without LiCl-induced Wnt pathway activation, in A549 and H1299 cell lines. *p < 0.05, **p < 0.01, and ***p < 0.001 compared with sh-NC group. ^^^*p* < 0.05, ^^^^*p* < 0.01, and ^^^^^*p* < 0.001 compared with sh-TMEM33 group.

LUAD progression is influenced by the deregulation of various signaling pathways. To explore which signaling cascade is implicated in TMEM33 function, we profiled the activation status of 10 different signaling pathways in LUAD cells after TMEM33 silencing using luciferase reporter assay. Our results revealed that knocking down TMEM33 apparently decreased luciferase reporter activity in the Wnt/β-catenin pathway (Fig. 5A). Subsequently, this study explored how TMEM33 affected key molecules and target gene expression within the Wnt/β-catenin pathway. In this study, depleting TMEM33 markedly down-regulated β-catenin, c-Myc, and Cyclin D1 proteins in comparison with the control group. To confirm the involvement of this pathway, we applied Lithium chloride (LiCl) to activate the Wnt/β-catenin pathway. LiCl promoted β-catenin, c-Myc, and Cyclin D1 expression within control and TMEM33-silencing cells (Fig. 5B). We observed that LiCl treatment could promote cell proliferation (Fig. 5C), invasion (Fig. 5D), and mesenchymal markers (Vimentin and N-cadherin) expression (Fig. 5E) in both control and TMEM33-silencing cells, while TMEM33-silencing suppressed these features compared to the control cells. Moreover, spheroid formation in both control and TMEM33-silencing cells was also enhanced by LiCl (Fig. 5F). These findings indicate that TMEM33 impacts the LUAD cell malignancy through modulating the Wnt/β-catenin pathway.

### TMEM33 silencing suppresses LUAD cell proliferation and migration in vivo

**Figure 6:**
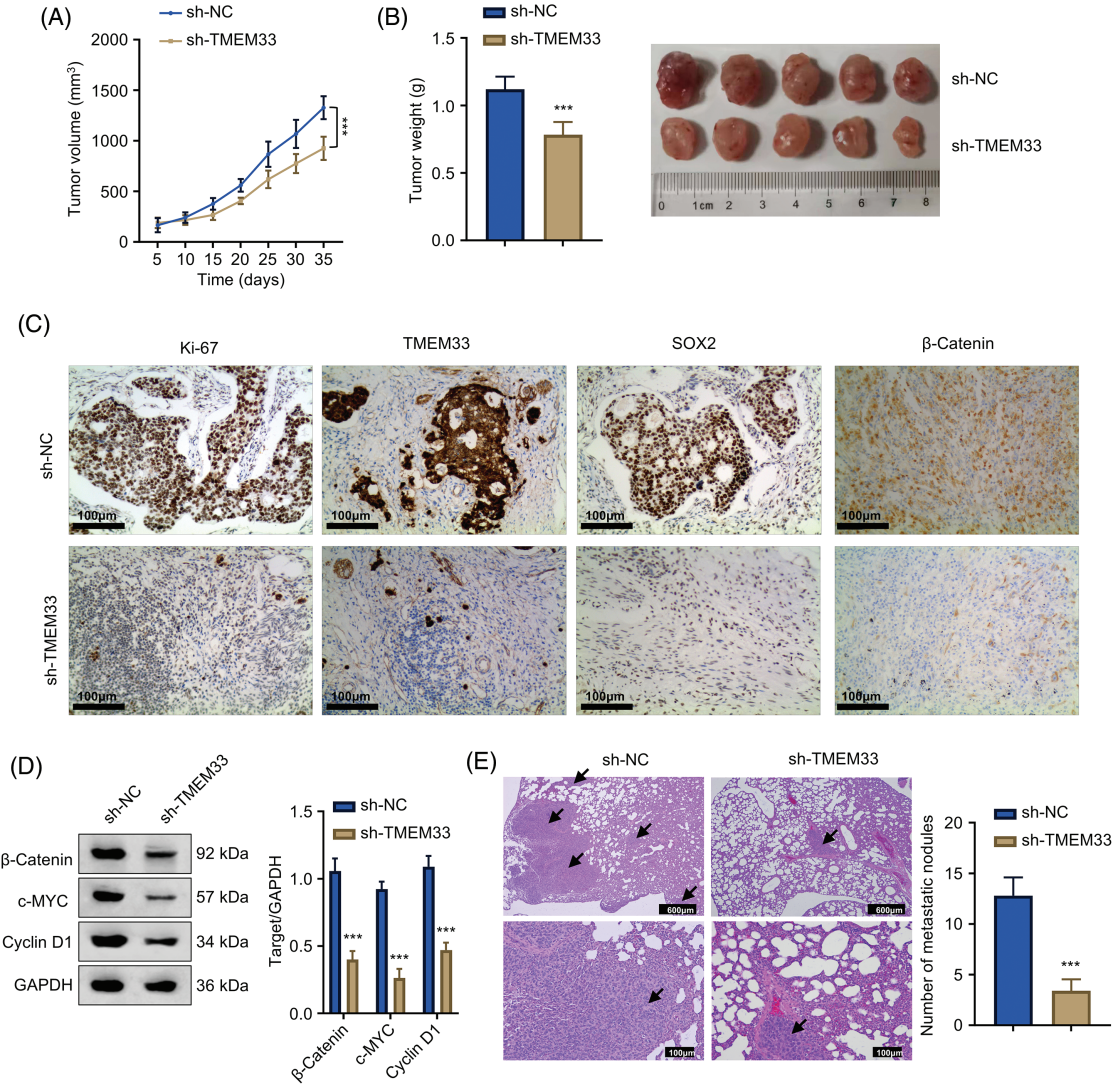
Knockdown of TMEM33 impedes LUAD cell proliferation and metastasis *in vivo*. (A) Tumor size was monitored at five-day intervals over a period of 35 days following the injection of A549 cells, with comparisons made between those with TMEM33 knockdown and those without. (B) Grafted tumors were taken 5 weeks after injection to analyze the weight of transplanted tumors. (C) Protein expression of Ki-67, TMEM33, and SOX2 in transplanted tumor tissues was detected by immunohistochemistry (scale bar = 50 μm). (D) Protein expression of β-catenin, c-MYC, and Cyclin D1 in transplanted tumors of control and knockdown TMEM33 groups was detected by immunoblotting. (E) HE staining to detect and count metastatic lesions in a LUAD metastasis model (scale bar = 600 or 100 μm. Arrows indicate lung metastatic nodules). ***p < 0.001.

For assessing TMEM33’s potential effect *in vivo*, a mouse xenograft model of LUAD was established utilizing A549 cells after sh-NC or sh-TMEM33 transfection. As a result, xenograft tumor weight and volume significantly decreased in the sh-TMEM33 group relative to the control group (Fig. 6A,B). Furthermore, Ki-67, TMEM33, and SOX2 protein expression in tumors from the sh-TMEM33 group exhibited a marked decrease, indicating a decrease in tumor aggressiveness and stemness (Fig. 6C). The immunoblotting analysis also revealed that β-catenin, c-Myc, and Cyclin D1 expression apparently decreased within tumors from the sh-TMEM33 group (Fig. 6D). Additionally, in a metastasis model induced via tail vein injection, the metastatic nodule number within the lungs was reduced in the sh-TMEM33 group, confirmed through the enumeration of metastatic foci (Fig. 6E). These findings further corroborate that TMEM33 is required for the malignant behavior of LUAD cells *in vivo* by modulating the Wnt/β-catenin pathway.

## Discussion

Lung cancer incidence and death rates are on the rise each year, underscoring the urgent necessity to discover biomarkers that can assist in managing and treating this malignancy [22,23]. Accumulating evidence also suggested that genes that support cancer development, like TMEM33, could be useful as indicators for diagnosing and treating cancer patients [7,8]. Our study carried out a comprehensive evaluation of how TMEM33 affects lung cancer cell proliferation, invasion, and stemness, highlighting the potential implication of the miR-214-3p/TMEM33 pathway in LUAD progression.

Previous findings suggest that TMEM33 has an important effect on cancer development [24,25]. However, its specific functions in LUAD are still unknown. In cervical cancer, TMEM33 could potentially be leveraged as the marker used to predict prognosis and immune infiltration, with its increased expression associated with higher cell proliferation rates [7,8]. Our analysis of TCGA database data revealed a significant upregulation of TMEM33 in LUAD tissues, which was further validated in the clinical samples. As a result, we initiated various investigations to explore the influence of varying TMEM33 expression levels within LUAD cells and elucidate specific molecular mechanisms at play. Moreover, diverse expression patterns and regulatory pathways of miR-214-3p are extensively explored in different cancer types, including those affecting the intestine, cervix, esophagus, and liver [26–28]. This not only highlights its crucial role in carcinogenesis but also underscores the significance of studying miR-214-3p in LUAD.

Our study has shown that TMEM33 functions as the target gene controlled by miR-214-3p, where miR-214-3p directly regulates its expression, impacting the Wnt/β-catenin pathway. Although miR-214-3p’s exact role in LUAD is not fully understood, CircTADA2A can trap miR-214-3p and eukaryotic translation initiation factor 4A3 (EIF4A3) to boost mitogen-activated protein kinase 8 (MAPK8) levels, promoting invasion and migration in lung cancer cells [18]. Likewise, CircCPA4 has been shown to absorb miR-214-3p, leading to elevated TGFB-induced factor homeobox 2 (TGIF2) expression and lung cancer development [19]. Moreover, miR-214-3p has been related to the identification of cancer stem cells (CSCs) by targeting Yes-associated protein 1 (YAP1), suggesting a potential therapeutic strategy for squamous lung cancer [29]. Our results indicate that TMEM33 expression is increased in LUAD tissues and may be a valuable indicator for diagnosis and prognosis in LUAD patients. Considering that TMEM33 expression is influenced by various signals, including miR-214-3p, and is tied to LUAD prognosis, it may be the target for treating lung cancer.

While providing valuable insights into the oncogenic role of TMEM33 and its regulation by miR-214-3p within LUAD, there are some limitations in the present work. Larger patient cohorts and additional animal models are needed to validate the findings across diverse populations. Furthermore, the precise molecular interactions between TMEM33 and the Wnt/β-catenin pathway require further elucidation, and the role of modulating miR-214-3p in the Wnt/β-catenin pathway needs to be verified. Investigation into the potential roles of TMEM33 in other cancer hallmarks beyond proliferation, invasion, and stemness is warranted. Exploring the role of targeting the miR-214-3p/TMEM33 pathway in cancer treatment could open new treatment avenues.

**Figure 7:**
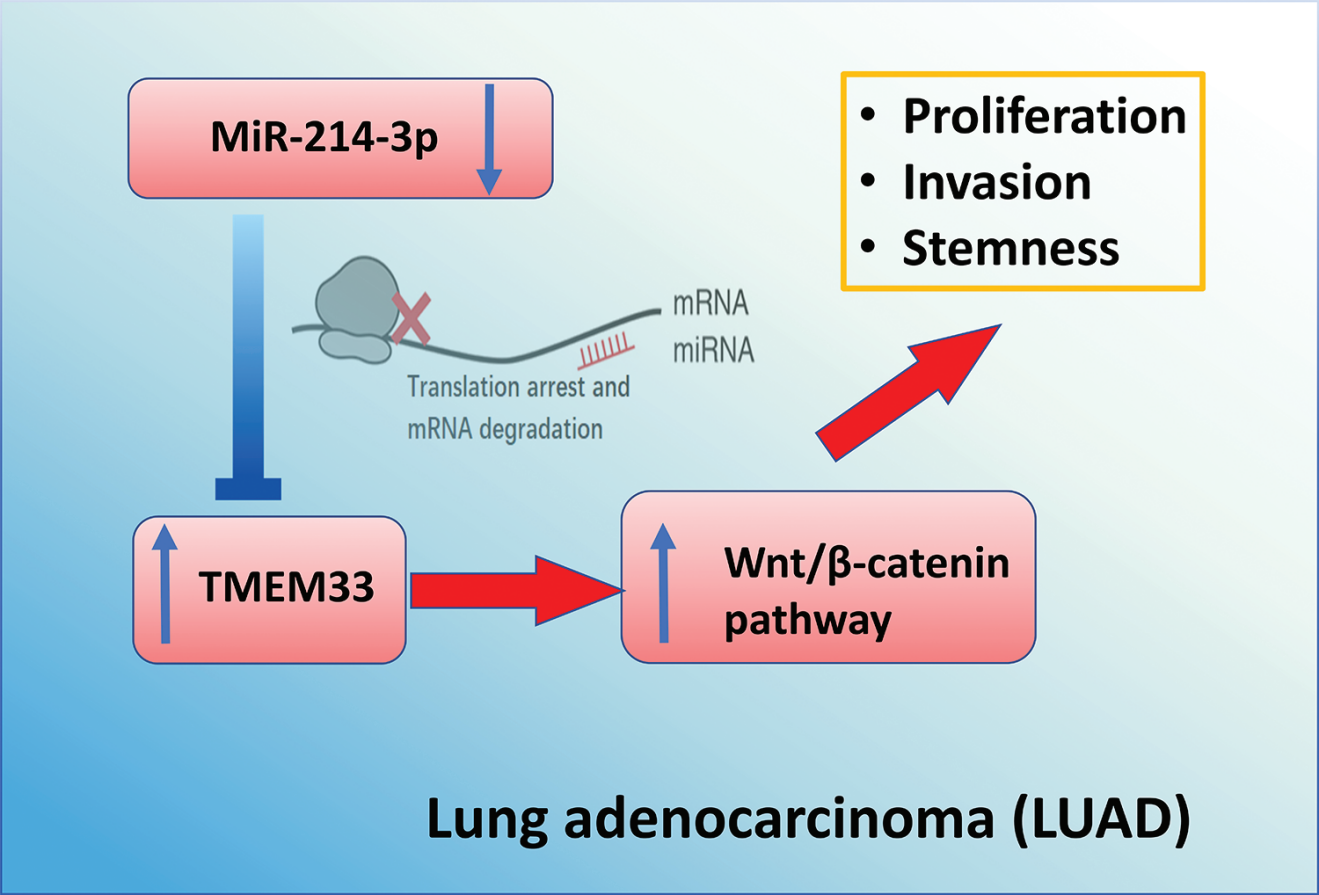
Schematics of the mechanism by which miR-214-3p/TMEM33 axis impinges on the progression of LUAD. Through the canonical process of mRNA interference, miR-214-3p modulates the levels of TMEM33, thereby regulating the growth, invasion, and stem cell characteristics of LUAD cells through targeting Wnt signaling. These findings highlight the opportunity of manipulating the miR-214-3p/TMEM33 axis as a potential strategy for LAUD management.

To summarize, our study has revealed a molecular cascade that includes miR-214-3p, TMEM33, and the Wnt pathway. By mRNA interference, miR-214-3p modulates TMEM33 expression, thereby regulating the growth, invasion, and stem cell characteristics of LUAD cells through targeting Wnt signaling (Fig. 7). These results suggest that manipulating the miR-214-3p/TMEM33 axis may be a potential strategy for LAUD management.

## Conclusion

In summary, our study demonstrated that TMEM33 functions as an oncogene in LUAD, promoting tumor progression through the Wnt/β-catenin signaling pathway. TMEM33 is negatively regulated by miR-214-3p, and its knockdown significantly reduces cancer cell proliferation, invasion, and stem-like properties both *in vitro* and *in vivo*. These findings highlight the therapeutic potential of targeting the miR-214-3p/TMEM33 axis in LUAD treatment.

## Data Availability

The data in this study is available upon email request to the corresponding author.
